# Targeting of Arenavirus RNA Synthesis by a Carboxamide-Derivatized Aromatic Disulfide with Virucidal Activity

**DOI:** 10.1371/journal.pone.0081251

**Published:** 2013-11-20

**Authors:** Claudia S. Sepúlveda, Cybele C. García, Jesica M. Levingston Macleod, Nora López, Elsa B. Damonte

**Affiliations:** 1 Laboratorio de Virología, Departamento de Química Biológica, Facultad de Ciencias Exactas y Naturales, Universidad de Buenos Aires, IQUIBICEN-Consejo Nacional de Investigaciones Científicas y Técnicas (CONICET), Buenos Aires, Argentina; 2 Centro de Virología Animal (CEVAN), Instituto de Ciencia y Tecnología Dr. César Milstein, CONICET, Buenos Aires, Argentina; George Mason University, United States of America

## Abstract

Several arenaviruses can cause severe hemorrhagic fever (HF) in humans, representing a public health threat in endemic areas of Africa and South America. The present study characterizes the potent virucidal activity of the carboxamide-derivatized aromatic disulfide **NSC4492**, an antiretroviral zinc finger-reactive compound, against Junín virus (JUNV), the causative agent of Argentine HF. The compound was able to inactivate JUNV in a time and temperature-dependent manner, producing more than 99 % reduction in virus titer upon incubation with virions at 37°C for 90 min. The ability of **NSC4492**-treated JUNV to go through different steps of the multiplication cycle was then evaluated. Inactivated virions were able to bind and enter into the host cell with similar efficiency as control infectious particles. In contrast, treatment with **NSC4492** impaired the capacity of JUNV to drive viral RNA synthesis, as measured by quantitative RT-PCR, and blocked viral protein expression, as determined by indirect immunofluorescence. These results suggest that the disulfide **NSC4492** targets on the arenavirus replication complex leading to impairment in viral RNA synthesis. Additionally, analysis of VLP produced in **NSC4492**-treated cells expressing JUNV matrix Z protein revealed that the compound may interact with Z resulting in an altered aggregation behavior of this protein, but without affecting its intrinsic self-budding properties. The potential perspectives of **NSC4492** as an inactivating vaccinal compound for pathogenic arenaviruses are discussed.

## Introduction

The *Arenaviridae* family consists of a unique genus, Arenavirus, comprising at least 23 recognized species [1]. Arenaviruses are classified into two distinct groups: Old World (OW) and New World (NW), both of which include important human pathogens. Within the OW group, Lassa virus causes severe hemorrhagic fever (HF) in West Africa and is associated with significant morbidity and mortality in humans. The prototypic arenavirus, lymphocytic choriomeningitis virus (LCMV), usually associated with transient asymptomatic or mild illness, has been also implicated as the etiologic agent of aseptic meningitis in humans [2]. The NW group includes Junin (JUNV), Machupo, Chapare, Guanarito and Sabiá viruses, which are well-known agents causing HF in different regions of South America. Because of their ability to establish chronic infections in certain rodent hosts, arenaviruses are typically associated with emerging endemic disease [3].

Arenaviruses are enveloped viruses; their genome is composed of two single-stranded molecules of RNA called L (ca 7.1 kb) and S (ca 3.4 kb), both exhibiting an ambisense coding strategy. The S segment encodes the nucleocapsid protein (NP) and the envelope glycoprotein precursor (GPC). GPC is processed post-translationally yielding a mature glycoprotein complex formed by three subunits that remain non covanlently linked: the signal peptide SSP, the external receptor-binding GP1 and the transmembrane fusion GP2 protein [4-6]. The L segment encodes the RNA-dependent RNA polymerase L and a small protein called Z. Several lines of evidence indicate that Z is essential for viral particle assembly and release [7-10]. Indeed, like the matrix protein of other enveloped viruses, Z protein self-associates into oligomeric forms, binds to cellular membranes, displays self-budding activity and is able to mediate the incorporation of NP and the envelope viral glycoproteins into virus-like particles (VLPs) [8,9,11-14]. 

Among the recognized pathogenic arenaviruses, only LASV and JUNV generate periodic annual outbreaks of Lassa fever and Argentine HF, respectively, and represent the main health threat in the family. At present, options for patient treatment are very limited. Immune plasma transfusion is the current and effective therapeutic intervention against Argentine HF, reducing the mortality to less than 1% when administered up to 8 days post-onset of symptoms [15,16]. However, the immune passive therapy presents several drawbacks such as the development of a late neurological syndrome by 10% of treated patients [15,16], the risk of transfusion-associated diseases, and the difficulties for supply and maintenance of adequate stocks of immune plasma. For Lassa fever patients, administration of ribavirin (RIB), a guanosine analogue that exhibits a broad spectrum of antiviral activity against RNA viruses, has proved to be efficient in reducing fatality rates from 50 % to 5-9 % when given before day 6 in the course of illness [17]. However, RIB therapy presents a series of disadvantages including a high level of undesirable secondary reactions such as thrombocytosis and anemia [18], the insufficient drug availability and high cost in developing countries, and finally the lack of knowledge about the mechanisms by which RIB exerts its anti-arenaviral action [19,20]. Furthermore, the clinical evaluation of RIB in other arenavirus HF patients did not show efficacy in reducing mortality [15]. With respect to preventive vaccination, the live attenuated JUNV vaccine Candid 1 was generated in the early 1990s. The immunization of at-risk population in Argentina with Candid 1 showed a protective efficacy greater or equal to 84 % without adverse effects and has led to a consistent reduction of Argentine HF in recent years [21,22]. This vaccine was licensed for use exclusively in Argentina and currently there is no evidence of cross protection against the other HF-causing arenaviruses. For LASV, the situation appears to be more complex and although there has been much effort to develop vaccines against Lassa fever none has been effective to warrant clinical trials [23]. Thus, alternative strategies for treatment and prevention against pathogenic arenaviruses are needed. 

In an effort to analyze alternative therapeutic molecules, we have previously shown that antiretroviral compounds with diverse chemical structures, which target to the zinc finger motifs in the human immunodeficiency virus type 1 (HIV-1) nucleocapsid protein [24,25], also display antiviral and virucidal activity against arenaviruses [26-29]. Moreover, the carboxamide-derivatized disulfide **NSC4492** was demonstrated to exhibit a moderate antiviral activity as well as a very potent virucidal effect against JUNV and other arenaviruses, including the non-pathogenic Tacaribe (TCRV) virus [30]. Here, we have further investigated the mechanism of JUNV inactivation by **NSC4492** and analyzed the *in vitro* inhibitory activity of this compound. The potent virucidal effect exhibited by **NSC4492** on arenaviruses points this compound as a promising tool not only for prophylactic therapy but also for its potential use in the production of inactivated virus vaccines. 

## Materials and Methods

### Compound

The carboxamide-derivatized aromatic disulfide **NSC4492** was provided by the National Cancer Institute, Frederick, MD, USA. Stock solutions at a concentration of 100 mM were prepared in dimethylsulfoxide (DMSO). Working solutions of **NSC4492** were prepared by dilution of the 100mM stock in the appropriate culture medium. 

### Cells and viruses

Vero cells were grown as monolayers in Eagle’s minimum essential medium (MEM, Invitrogen-Life Technologies) containing 5 % inactivated bovine serum and 50 µg/ml gentamycin. Maintenance medium (MM) consisted of MEM supplemented with 1.5 % bovine serum. CV1 cells and BSR cells (a BHK-21 clone) were grown in Dulbecco's MEM (D-MEM, Invitrogen-Life Technologies) and Glasgow MEM (G-MEM, Invitrogen - Life Technologies, USA), respectively, supplemented with 2 mM glutamine, 10% fetal bovine serum (FBS) and penicillin (100 U/ml)-streptomycin (100 µg/ml) (Invitrogen-Life Technologies).

All experiments were performed using the attenuated strain IV4454 of JUNV [31]. Virus stocks were prepared in Vero cells and titrated by plaque assay on the same cells.

### Virus inactivation

For virus inactivation assays, treatment of JUNV with **NSC4492** was carried out by mixing an aliquot of a viral suspension containing approximately 1x10^6^ plaque-forming units (PFU) with the same volume of the appropriate **NSC4492** working solution followed by incubation at the temperature and times indicated. As control, an equivalent aliquot of the virus suspension was incubated in parallel with MM under the same conditions. Then, samples were chilled, further diluted with MM and used to determine the remaining viral titer by plaque assay. In a similar inactivation assay, we have previously determined that the incubation of virus with MM containing DMSO 1:5000-1:100000 (dilutions of solvent corresponding to the working solutions of **NSC4492**) did not affect JUNV infectivity. Incubation times required to produce 50, 90 and 99 % reduction in virus titer, T-50, T-90 and T-99, respectively were calculated from the inactivation kinetics curve. 

### Virus adsorption and internalization

For evaluation of adsorption, Vero cells were infected with JUNV suspensions previously treated or not with **NSC4492**, at a multiplicity of infection (m.o.i.) of 1 PFU/cell. The indicated values of m.o.i. always refer to starting PFU activity previous to inactivation. After 5 and 60 min of adsorption at 4°C, cells were extensively washed with cold PBS and total RNA was extracted by using RNeasy Mini Kit (Qiagen) according to the manufacturer´s instructions. To monitor cell-bound viral RNA, cDNA was generated from purified RNA by using murine reverse transcriptase M-MLV (Invitrogen-Life Technologies) and random primers. This cDNA was amplified by real time PCR using SYBRGreen (Roche) detection and specific primers for the Z gene. Actin mRNA was amplified with the corresponding gene specific primers. The primer sequences and reaction conditions for real time RT-PCR were previously reported [32]. Average viral RNA Ct values were normalized to the average Ct values of actin and ΔΔCt based fold-change calculations were set relative to untreated-virus infected cells using Bio-Rad iQ5 2.1 software. 

For quantification of internalized virus, after adsorption as above cells were further incubated in MM at 37°C for 1 h. Then, culture media were discarded; cells were washed with PBS and treated with a solution of 1 mg/ml proteinase K (Invitrogen-Life Technologies) for 45 min at 4°C. Proteinase K was inactivated with PBS- 0.2% bovine serum albumin (BSA) containing 2 mM phenylmethylsulfonyl fluoride, detached cells were transferred into a tube and washed twice with PBS-0.2 % BSA by low-speed centrifugation. Total RNA was extracted from pelleted cells and internalized viral RNA was evaluated by real time RT- PCR, as above.

### Virus uncoating

Vero cells grown in coverslips were infected with **NSC4492**-treated or untreated JUNV at a m.o.i of 10 PFU/cell. After 1 h of adsorption at 4°C, cells were washed and incubated at 37°C in MM with the addition -or not- of concanamycin A at a final concentration of 50 nM. At the indicated times, cells were fixed in 4% paraformaldehyde for 10 min at 37 °C, then incubated with 20 mM NH_4_Cl for 10 min at 37 °C and permeabilized with PBS 0.5 % Triton X-100 for 15 min at room temperature. Cytoplasmic NP immunofluorescence staining was carried out with the monoclonal antibody (mAb) SA02-BG12 [33], followed by Alexa Fluor 488-goat anti-mouse IgG (Invitrogen-Life Technologies) as secondary antibody. After a final washing with PBS, cells were mounted in a glycerol solution containing 1,4-diazabicyclo[2, 2, 2]octane (DABCO) and visualized under confocal fluorescence microscope.

### Virus macromolecular synthesis

To analyze virus RNA synthesis, Vero cells were infected with NSC4492-treated or untreated JUNV at a m.o.i of 1 PFU/cell. At 1, 2.5, 5, 7 and 12 h p.i, total RNA was extracted by using RNeasy Mini Kit (Qiagen) and employed for cDNA synthesis by using M-MLV (Invitrogen-Life Technologies, USA) and a genomic sense JUNV GPC-specific primer. The cDNA was further amplified using GPC-specific primers by real time PCR as above. Average viral RNA Ct values were normalized to the average Ct values of actin and ΔΔCt based fold-change calculations for untreated and treated- virus infected cells were set relative to the value of untreated-virus infected cells at 1 h p.i., defined as 1, using Bio-Rad iQ5 2.1 software. 

To determine viral protein expression, Vero cells grown in coverslips were infected with **NSC4492**-treated or untreated JUNV (m.o.i. 1 PFU/cell). At 16 h p.i., cells were fixed and processed for cytoplasmic and membrane immunofluorescence. For NP cytoplasmic staining, cells were fixed in methanol for 10 min at -20°C and then incubated with the mAb SA02-BG12, followed by fluorescein isothiocyanate (FITC)-goat anti-mouse IgG (Sigma Aldrich Co). For membrane staining, cells were fixed in 4% paraformaldehyde for 10 min at 37 °C and then incubated with 20 mM NH_4_Cl for 10 min at 37 °C. Then, cells were incubated with mAb QC03-BF11 for GPC/GP1 [33], followed by the FITC-labeled IgG as secondary antibody. In both cases, after a final washing with PBS, cells were stained with Evans Blue and mounted in DABCO.

### VLP purification and Western blotting

Approximately 4 x 10^5^ BSR cells grown in a 12-well dish were transfected with (amounts per well) 1 µg of plasmid pJUNV Z-HA, expressing an HA-tagged version of JUNV Z (JUNV Z-HA) along with 1 µg of plasmid pCMV-T7pol, which expresses the bacteriophage T7 RNA polymerase [34]. At 4 h post-transfection supernatants were removed, cells were washed twice with PBS, and supplemented with G-MEM 2% FBS containing or not **NSC4492** at a final concentration of 25 µM. Control cultures were supplemented with medium plus the corresponding volume of DMSO. Following incubation at 37°C for 48 h, culture supernatants were harvested and cell monolayers were lysed in non-reducing SDS-PAGE sample buffer (Invitrogen Life-Techcnologies). VLPs were purified from the cell culture supernatants by ultracentrifugation through 20% (wt/vol) sucrose cushions at 34,000 rpm for 2 h at 4°C in a Beckman SW 50.1 rotor. Purified VLPs, resuspended in nonreducing sample buffer, and cellular lysates were resolved by SDS-PAGE in gels containing 12% polyacrylamide and then transferred to nitrocellulose membranes. Blots were probed with a rabbit anti-HA polyclonal antibody (Santa Cruz Biotechnology) for 2 h at 37°C followed by incubation with horseradish peroxidase-conjugated anti-rabbit secondary antibody (Jackson ImmunoResearch) according to the supplier’s specifications. Detection was achieved by enhanced chemiluminescence, using SuperSignal West Pico chemiluminescent substrate (Thermo Scientific). Quantification of protein bands were carried out by densitometry using ImageJ software [35]. To normalize the amount of Z to the amount of actin in cell extracts, blots were stripped and then reprobed with an anti-actin primary antibody (Sigma-Aldrich Co), followed by enhanced chemiluminescence and quantification by densitometry, as above.

## Results

### JUNV inactivating activity of NCS4492: Time and temperature dependence

**Figure 1:**
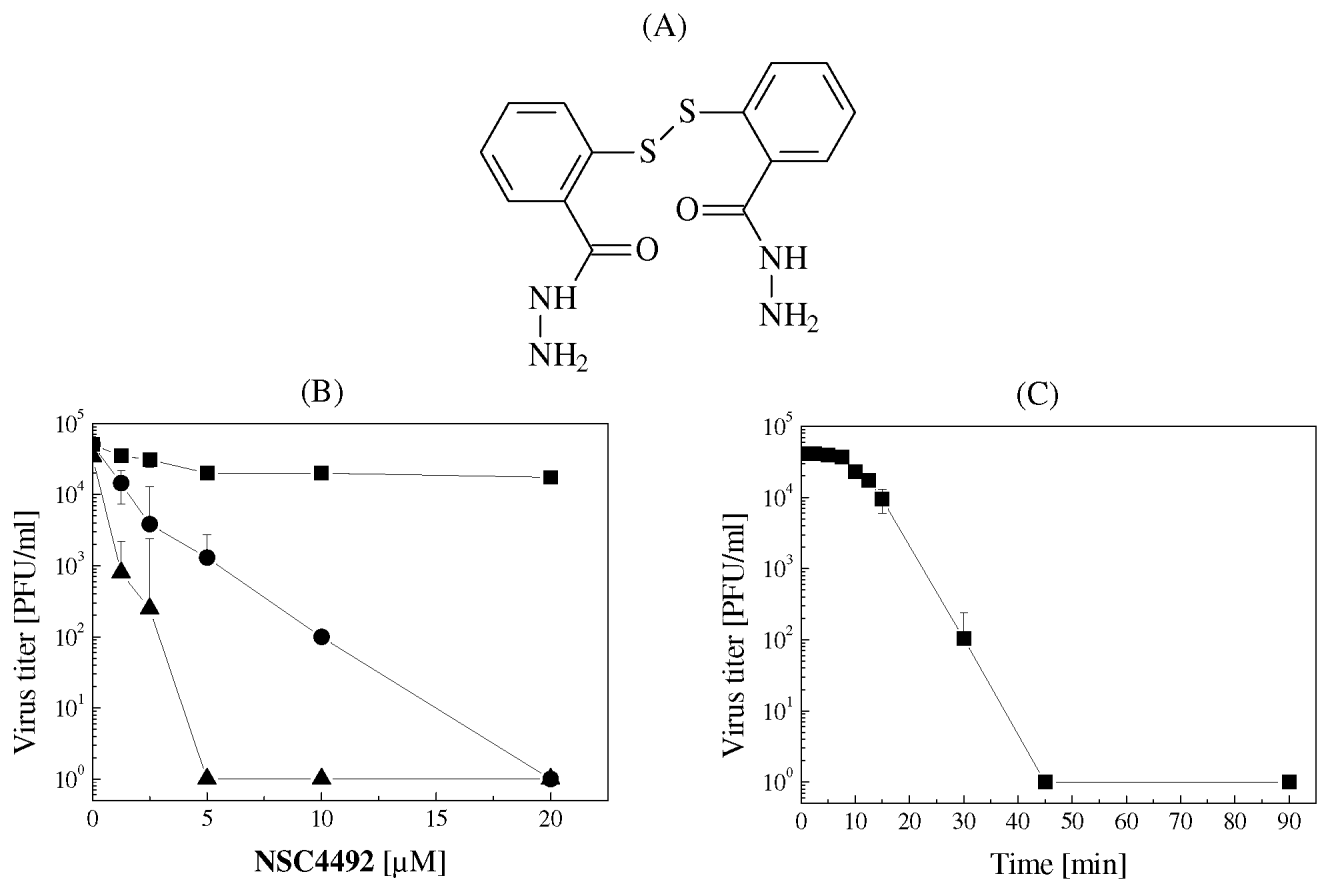
Time and temperature dependence of NSC4492 inactivating activity on JUNV. (A) Chemical structure of **NSC4492**. (B) Suspensions containing 1 x 10^6^ PFU of JUNV were incubated with increasing concentrations of **NSC4492** at 4°C (■), 25°C (●) or 37°C (▲) for 90 min. Then, the remaining infectivity was titrated by plaque assay in Vero cells. (C) Suspensions containing 1 x 10^6^ PFU of JUNV were incubated with 10 µM **NSC4492** at 37°C. At the indicated times, the remaining infectious virus was determined as in (B). Each value represents the mean of triplicate assays ± standard deviation (SD).

Previously, we performed a screening of a panel of aromatic disulfides and found that the carboxamide-derivatized **NSC4492** (Figure 1A) is a very potent virucidal agent against two closely related NW arenaviruses, TCRV and JUNV, with inactivating concentration 50% (IC_50_) values in the range of 0.2-0.7 µM [30]. To further characterize the inactivating properties of **NSC4492**, the temperature dependence of its biological effect was analyzed by incubation of JUNV in the presence of increasing concentrations of **NSC4492** at 4, 25 or 37°C (Figure 1B). The results showed a very weak inactivating effect at 4°C, with about 40% remaining infectivity at concentrations of the compound as high as 20 µM. The inactivating effect was drastically enhanced when virus treatment was carried out at 25°C, being maximal upon incubation at 37°C (Figure 1B), indicating that the interaction between **NSC4492** and virions is temperature dependent. Next, the kinetics of JUNV inactivation by **NSC4492** was evaluated using the optimal temperature and compound concentration established from Figure 1B, 37°C and 10 µM. A very significant loss of infectivity was observed as early as 10-15 min after addition of the compound to the virus suspension since the value of T-50 obtained from data shown in Figure 1C was 10.5 ± 1.8 min. The virucidal effectiveness of the disulfide was further evidenced by the strong inactivation produced after 30 min of incubation, with more than 99 % reduction of viral infectivity and values of T-90 and T-99 of 23.0 ± 3.9 and 29.4 ± 0.3 min, respectively. Finally, complete inactivation of JUNV with more than 4 log reduction in virus titer was observed after 45 min of treatment. These results lead us to adopt incubation conditions assessing potent virus inactivation (10µM **NSC4492** for 90 min at 37°C) for the following experiments.

### Mode of inactivation of JUNV virions by NSC4492

**Figure 2:**
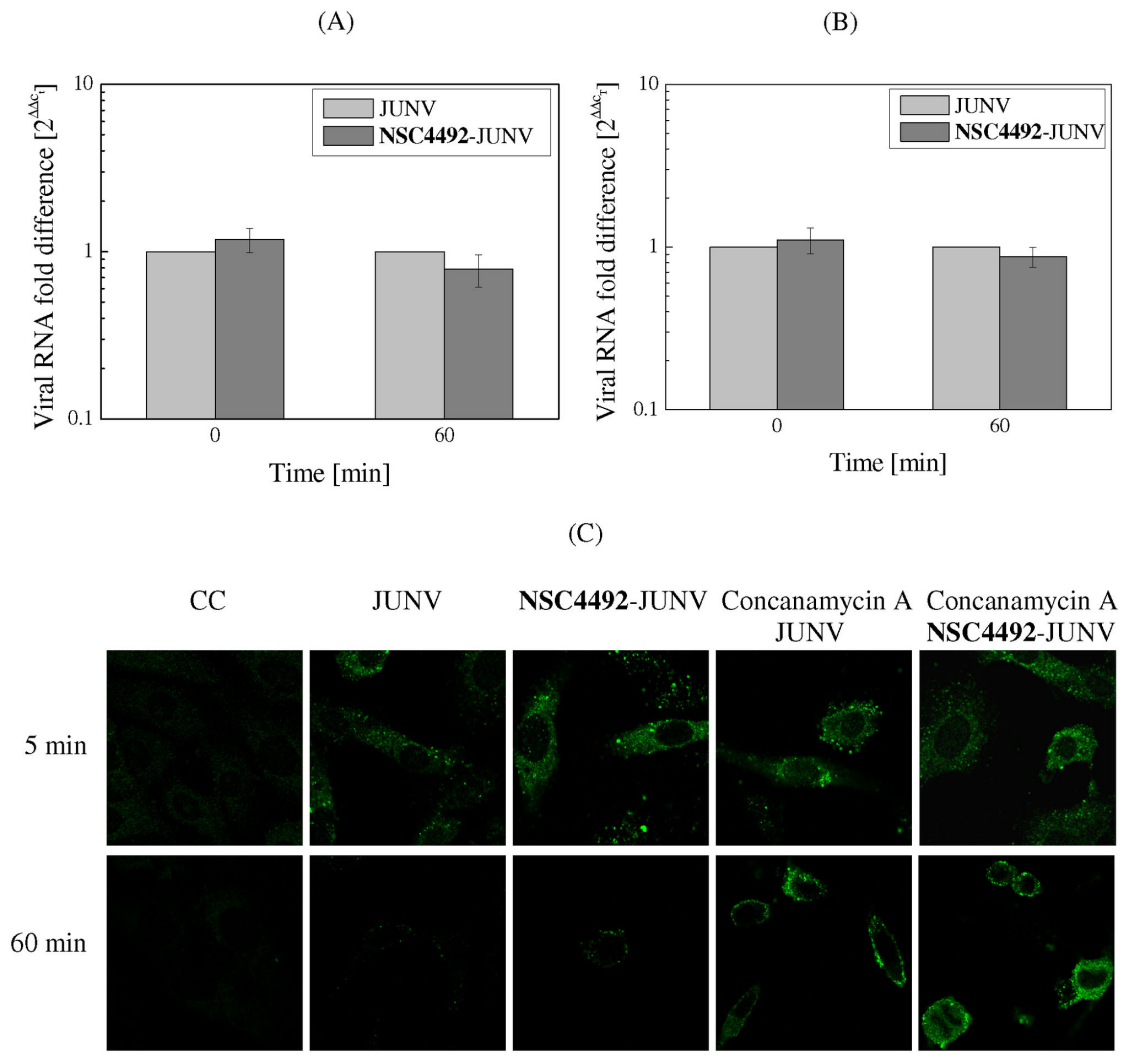
Entry of JUNV particles treated with NSC4492. (A) *Binding*. JUNV treated -or not- with 10 µM **NSC4492** for 90 min at 37°C was adsorbed at 4°C to Vero cells. Immediately after inoculation (time 0) or 60 min later, the inoculum was removed, total RNA was extracted and the amount of cell-bound viral RNA was determined by real-time RT-PCR using JUNV Z gene specific primers and cellular *actin* amplification for normalization. (B) *Internalization*. Vero cells were inoculated with JUNV inactivated or not with **NSC4492** as in (A). After adsorption for 1 h at 4°C followed by removal of the inoculum, cells were shifted to 37°C. At the indicated times, non internalized virus was removed by treatment with proteinase K, total RNA was extracted and real-time RT-PCR was performed as in (A) to determine the relative amount of internalized viral RNA. Results in (A) and (B) are expressed as fold difference of viral RNA level in cells infected with **NSC4492**-treated JUNV as compared to the corresponding untreated JUNV, set as 1. The values are averages of duplicate independent experiments ± SD. (C) *Uncoating*. Vero cells grown in coverslips were mock-infected or infected at 4°C with JUNV suspensions previously treated or not with **NSC4492**. Unadsorbed virus was removed and cells were supplemented with MM containing or not concanamycin A and incubation proceeded at 37°C for 5 or 60 min. Then, cells were processed to detect NP by IF staining. Representative cells of all fields in each sample are shown in the figure. Magnification: 600X plus digital zoom 2.5X.

As a first approach to elucidate the mechanism of JUNV inactivation by the disulfide **NSC4492**, we analyzed the effect of the compound on virus entry. To this end, the binding ability of **NSC4492**-inactivated virions to the cell membrane was first evaluated. Vero cells were inoculated with JUNV previously treated or not with **NSC4492** and the amount of cell-bound JUNV RNA that remained after extensive washing of the cell monolayers was quantified by real time RT- PCR. As shown in Figure 2A, treatment with **NSC4492** did not alter the level of cell-associated viral RNA detected at the beginning (0 min) of the adsorption step. Moreover, similar levels of viral RNA were observed at 60 min post- inoculation with either treated or untreated virions, suggesting that the receptor binding capacity of inactivated JUNV is comparable to that of control infectious particles. 

Next, a virus internalization assay was employed to analyze the effect of **NSC4492** on virion uptake. Briefly, JUNV previously treated -or not- with **NSC4492** was adsorbed to Vero cells for 1 h. Following incubation at 37°C to allow virus entry, cell monolayers were treated with proteinase K to remove adsorbed but not internalized virus, and intracellular JUNV RNA was quantified by real time RT- PCR. The results showed levels of viral RNA in cell monolayers inoculated with **NSC4492**-treated JUNV that were comparable to those in untreated JUNV-infected cells (Figure 2B). These results clearly demonstrated that **NSC4492** did not impair the uptake of JUNV into the host cells.

To further assess the effect of **NSC4492** on virus entry, we examined the ability of treated virus to exit the endosomal compartment and release the viral ribonucleoprotein into the cytoplasm. Cells were inoculated with untreated or **NSC4492**-treated virus and the presence of virions into cytoplasmic endosomal vesicles was detected by IF staining of NP. As control, untreated virus-infected cells were incubated with concanamycin A, a specific inhibitor of vacuolar-type ATPase activity that has been reported to raise endosomal pH of enveloped virus-exposed cells thus preventing membrane fusion [36,37]. As expected, cells infected with untreated virus showed a decreased amount of NP after 60 min of incubation at 37°C, as compared with that observed immediately after adsorption (Figure 2C), indicating that virion uncoating occurred and nucleocapsids were released from cellular endosomes after fusion. Cultures infected with **NSC4492**-treated virions revealed a pattern of NP immunofluorescence that was comparable to that observed for control untreated virus at 0 and 60 min of infection. In contrast, blockage of virus uncoating was visualized in cultures infected with control virus and treated with concanamycin A: a pattern of bright intracellular NP staining was observed at both times post-infection, with a strong accumulation of fluorescence in the perinuclear zone after 60 min of internalization (Figure 2C). When cells infected with **NSC4492**-inactivated JUNV were treated with concanamycin A, a similar pattern of NP fluorescence retention in the perinuclear region was detected (Figure 2C), indicative that the uncoating of inactivated virions is dependent on endosomal acidification, similar to untreated virus and in accordance with the penetration mechanism reported for JUNV [38,39]. Altogether, these results suggested that virion uncoating from the endosomal compartment was not impaired by treatment of JUNV with **NSC4492.**

**Figure 3:**
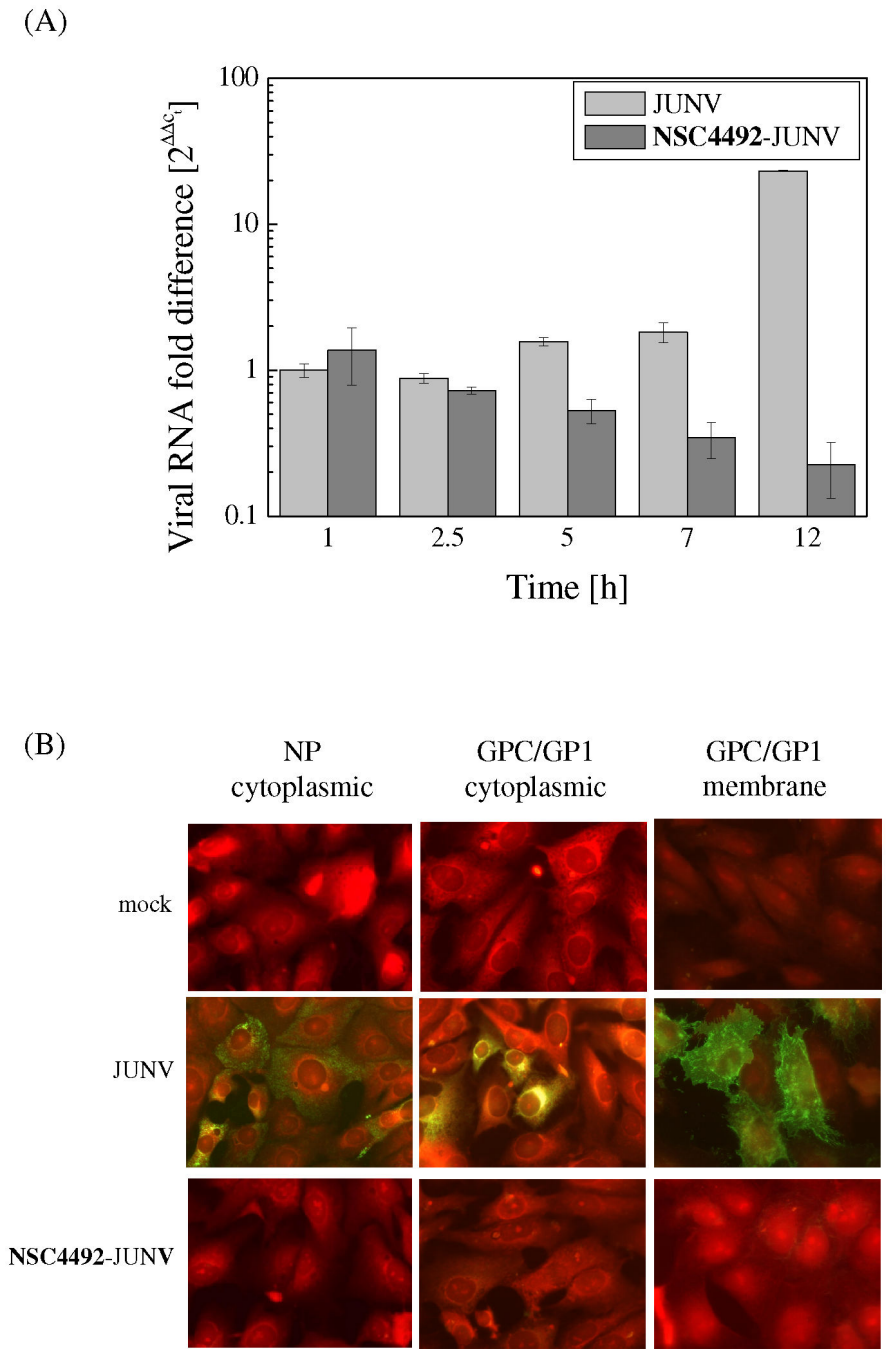
Analysis of viral macromolecule synthesis in cells infected with NSC4492-inactivated JUNV. (A) *RNA synthesis*. Vero cells were infected with JUNV previously treated or not with **NSC4492**. Total cellular RNA was extracted at the indicated h p.i. and viral RNA was quantified by real-time RT-PCR using primers specific for JUNV GPC. Results are expressed as fold difference of viral RNA level in cells exposed to untreated or **NSC4492**-treated JUNV as compared to viral RNA level at 1 h p.i. in cells infected with untreated JUNV, reference point defined as 1. The values are averages of duplicate independent experiments ± SD. (B) *Protein expression*. Vero cells were mock-infected or were inoculated with untreated or **NSC4492-**inactivated JUNV. At 16 h p.i., either cytoplasmic or membrane IF staining was performed to detect NP or GP1/GPC proteins. Magnification: 400X.

After penetration into the host cell, synthesis of viral RNA and proteins is the subsequent step in virus multiplication cycle. First, to analyze the possible targeting of **NSC4492** on viral RNA synthesis, the levels of JUNV RNA in cells infected with treated or untreated virions were comparatively quantified during 12 h of infection, the time required to complete the multiplication cycle of JUNV [40], by using real time RT- PCR. The amounts of viral RNA in cells infected with untreated or treated virions were calculated in comparison to the content of viral RNA in control infected cells at 1 h p.i., defined as 1. As seen in Figure 3A, the time course of JUNV RNA synthesis in control infected cells was in accordance with previous studies [32,41] with increasing levels of intracellular RNA from earlier to later times. At 1 h p.i., the content of viral RNA in cells infected with inactivated virions was similar to that in control infected cells, confirming that entry and uncoating are not affected and that the initial amount of RNA delivered into the cells through infection was similar. By contrast, the relative contents of viral RNA in cells infected with **NSC4492**-inactivated JUNV decreased with time, indicating that no new RNA molecules were synthesized whereas initially internalized viral RNA was degraded. The maximal difference between cells infected with control and treated virions was observed at 12 h p.i., when the peak in RNA synthesis was detected for untreated virions (Figure 3A). These results suggested that **NSC4492** impairs the ability of virions to direct viral RNA synthesis.

To further corroborate these results, the level of viral protein expression in infected cells was analyzed by indirect immunofluorescence. Both NP and GPC/GP1, the major structural arenavirus proteins, were clearly detected after 16 h of infection with untreated virus (Figure 3B). In contrast, no viral proteins could be observed either in the cytoplasm or the surface of cells infected with **NSC4492**-treated JUNV (Figure 3B). Altogether, these results demonstrated that treatment with **NSC4492** completely abolished the ability of JUNV particles to drive the biosynthesis of viral macromolecules within the host cell.

### Effect of NSC4492 on particle budding

**Figure 4:**
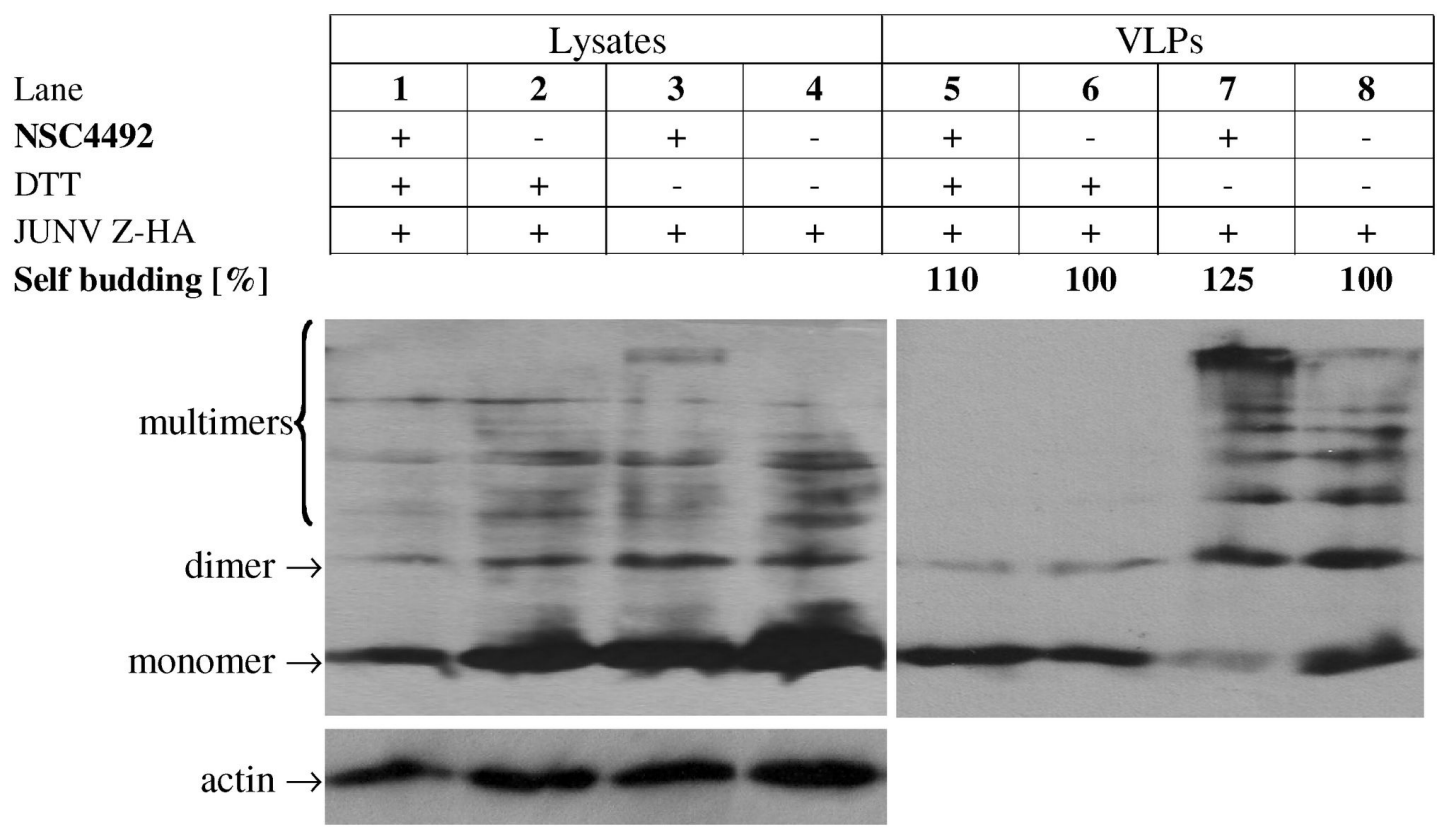
Effect of NSC4492 on Z-VLP production. BSR cells were transfected with plasmids pJUNV Z-HA and pCMV-T7pol, to express JUNV Z-HA protein. After 4 h, transfection medium was replaced with fresh medium with (+) or without (-) 25 µM **NCS4492** and incubation proceeded for 48 h. Aliquots of both cell extracts and VLPs purified from cell supernatants were boiled in SDS-PAGE sample buffer after addition (+) or not (-) of 0.1% dithiothreitol (DTT) and then analyzed by Western-blotting, using an anti-HA antibody. Protein bands were quantified by densitometry and the amount of Z in cell lysates was normalized to the amount of actin. Self-budding (%) corresponds to the ratio between Z protein detected in VLPs to total (VLPs plus lysates) Z protein. Bands consistent with monomeric, dimeric, and multimeric forms of Z are indicated.

After RNA and protein synthesis, virus assembly and budding represent the final step of JUNV multiplication cycle. It is known that arenavirus Z protein drives the cell surface budding of arenavirus particles in infected cells and is able to direct self-assembly and budding of VLPs, in the absence of any other viral protein [8,9,12,13,42,43]. To evaluate if **NSC4492** affects these late processes, we analyzed the effect of the compound on the ability of Z protein to drive VLP formation by employing a well-established VLP assay previously reported [12]. Briefly, mammalian cells were transfected with the plasmids pJUNV Z-HA and pCMV-T7pol to express only JUNV Z-HA followed by incubation of cells with or without **NSC4492** for 48 h. Then, VLPs were purified from cell supernatants, aliquots of both cell lysates and purified VLPs were resolved in SDS-polyacrylamide gels and Z protein was detected by immunoblotting (Figure 4). Reprobing of blots with an anti-actin antibody was performed to control for gel loading. Evaluation of the cell lysates revealed comparable levels of Z protein normalized to cellular actin in the presence or absence of the compound, both under reducing or non-reducing conditions (lanes 1 to 4), indicating that **NSC4492** did not substantially affect Z expression in transfected cells. With respect to VLP formation, the level of Z self-budding, calculated as the ratio between Z protein detected in VLPs to total Z protein (VLPs plus lysates), was not diminished by **NSC4492** (lanes 5 to 8). When samples were analyzed under reducing conditions, similar levels of Z as a monomer were detected in VLPs formed either in the presence or absence of the compound (lanes 5 and 6). By contrast, analysis under non-reducing conditions clearly showed an altered pattern of Z protein electrophoretic migration in VLPs released from **NSC4492**-treated cells compared to control samples, with decreased amount of Z monomer and increased amount of higher molecular weight multimers (lanes 7 and 8). Overall, these results suggested that **NSC4492** altered the capacity of Z protein to self-aggregate into oligomers, although this alteration did not impact on the ability of Z to drive VLP formation.

## Discussion

The studies here reported confirm previous results [30] about the potent virucidal activity of the disulfide **NSC4492**, a compound able to inactivate JUNV particles in the range of micromolar concentrations leading to total loss of infectivity at controlled experimental conditions. Our earlier studies also demonstrated the arenavirus-inactivating effect of another disulfide, the compound **NSC20625**, likely caused by interaction of the drug with the Z protein [28,29]. Moreover, **NSC20625** was shown to induce metal-ion ejection from purified LCMV Z protein, with the consequent loss of its native structure and stability [28]. In the present study, we further analyzed the mode of interaction of **NSC4492** with JUNV which results in loss of virion infectivity.

Aromatic disulfides, like **NSC4492**, display a well-known antiretroviral activity through its interaction with the retrovirus nucleocapsid (NC) protein zinc finger motifs [24,44,45]. Several studies demonstrated that treatment of virions with the compounds results in zinc ejection from the zinc fingers of NC protein with the consequent formation of multimeric aggregates by intra- or intermolecular NC cross-linkage [44,46-48]. These alterations within the viral core structure correlate with loss of virion infectivity by blockade of reverse transcription [47,49].

Sequence comparisons and structural studies revealed that three of the arenavirus proteins display zinc-binding motifs. The matrix Z protein contains a conserved RING motif that coordinates two zinc ions. The structural integrity of the RING is required for proper protein folding [50], and is essential for Z-mediated inhibition of viral RNA synthesis as well as for the interaction between Z and other viral and cellular proteins [12,14,51-54]. The transmembrane GP2 contains a zinc-binding domain consisting of two arrays of conserved Histidine and Cysteine residues that coordinate two zinc atoms. This motif has been implied in maintaining the structure and function of the envelope tripartite glycoprotein complex [55,56]. Finally, a CCHE zinc-binding site has been described in the C-terminal domain of the nucleoprotein NP, which is likely important for stabilizing the overall structure of the domain [57-60]. 

Based on these precedents, our hypothesis was that **NSC4492** might target the arenavirus Z protein and/or any of the viral proteins carrying zinc-binding motifs. We used different experimental approaches to identify the step of virus multiplication cycle as well as viral component/s targeted by the compound. To examine the possible effect of **NSC4492** on the arenavirus glycoprotein complex, the initial events leading to virus entry were evaluated. Our results showed that **NSC4492-**treated virions exhibit abilities to bind cell surface receptors, to penetrate and uncoat into the host cell that were comparable to those of infectious particles (Figure 2). In fact, the infection of cells with treated JUNV in the presence of concanamycin A evidenced that inactivated virions employed an endocytic route dependent on acid pH for membrane fusion similarly to control untreated virions. These results indicated that the biological functions of the envelope glycoprotein complex are not affected by treatment with **NSC4492**, implying that the zinc finger motif present in GP2 is likely not altered by the compound.

To test whether Z protein can be a target of **NSC4492**, we analyzed the effect of the compound on the ability of Z to drive particle assembly and budding. A change in the electrophoretic mobility pattern of Z protein was observed when Z-VLPs produced by **NSC4492**-treated cells were analyzed under non-reducing conditions (Figure 4). These results are consistent with previous findings showing that treatment of purified recombinant LCMV Z protein with the disulfide **NSC20625** induces the formation of high molecular weight Z multimers [28]. Nevertheless, the **NSC4492-**induced change in the profile of Z oligomers did not correlate with an impaired VLP formation, as no difference in the amounts of Z-VLPs released from either **NSC4492-**treated or control cultures was observed (Figure 4). These results indicated that the compound does not affect the intrinsic Z self-budding activity.

The crucial process affected by **NSC4492** in inactivated virions was viral RNA synthesis. Although the amount of viral RNA delivered to the cytoplasm at early times after uncoating (1 h in Figure 3A) is comparable between cells infected with control or treated JUNV, de novo synthesis of RNA is not driven by inactivated virions even at late times when one cycle of multiplication of JUNV is completed (12 h in Figure 3A). This strong inhibition of viral RNA synthesis in cells infected with inactivated JUNV suggests the possibility that **NSC4492** may target the nucleocapsid functionality. Arenavirus nucleocapsids functional for RNA synthesis are formed by association of genomic RNA with the L polymerase and many molecules of NP in an helicoidal structure. It is interesting to note that arenavirus L and NP proteins interact with each other and that this interaction is thought to have an important role during replication and transcription [53,61]. Since no zinc-binding motifs have been identified within the L protein so far, it is tempting to speculate that an effect of the compound on the zinc-binding domain of NP may account for the observed results. One possibility is that an **NSC4492**-induced alteration on NP may influence the NP-L interaction and, consequently, affect the biological function of both proteins. However, the possible targeting of **NSC4492** on the L protein cannot be ruled out.

Otherwise, it cannot be discarded that conformational changes of Z induced by the compound could be indirectly involved in the impairment of viral RNA synthesis observed in cells infected with **NSC4492**-inactivated JUNV. Several studies have reported that Z protein is able to modulate viral RNA synthesis through interaction with the L polymerase [14,53,62]. The structural change produced in Z by the disulfide may alter the binding affinity of Z to L leading to a blockade of the RNA polymerase activity. Further research is required to fully elucidate whether **NSC4492** is targeted to reactive motifs in one or more viral components to produce complete JUNV inactivation.

The susceptibility of different zinc-binding motifs to aromatic disulfides and other zinc finger-reactive compounds is still not fully understood. However, molecular modeling and experimental studies suggest that the impact of these compounds on zinc fingers depends on the interplay of multiple factors, such as binding affinity, ligand reactive proximity and sufficient redox properties to react with the cysteines and promote zinc ejection [63,64]. For instance, the Z protein-reactive disulfide **NSC20625** would not impact on other cellular RING proteins, such as the promyelocytic leukemia protein PML [28]. Thus, it is not surprising that differential structural features of arenavirus zinc-binding domains could be related with the apparent differential reactivity of **NSC4492** towards each of the arenavirus proteins. 

Due to its potent virucidal effect at low doses (Figure 1), **NSC4492** might be considered as a promising tool for prophylactic treatments aimed at limiting the spread of arenaviruses, considered as viral biowarfare agents [65]. Furthermore, based on the inability of **NSC4492-**inactivated JUNV particles to drive viral RNA and protein synthesis within the host cell with apparent preservation of viral glycoprotein functions, the compound may be envisaged as a good candidate for its use in the generation of inactivated virus vaccines. For retroviruses, diverse zinc finger-reacting compounds have been studied for their potential use in inactivated vaccine development. The 2,2’-dipyridyl disulfide, also known as aldithriol-2 (AT-2), as well as N-ethylmaleimide (NEM) were reported to inhibit virion infectivity by reacting with the nucleocapsid protein without altering the antigenic properties of the virus [66,67]. Similarly, preservation of the integrity of conformational epitopes in the viral envelope glycoprotein upon hantavirus inactivation by NEM has also been reported [68].

At present, the only approved vaccine for use against HF arenaviruses is the live attenuated Candid 1 vaccine, which has been licensed exclusively in Argentina after a clinical study in agricultural workers at risk [21,22]. While the Argentine HF vaccination program has been successful in reducing morbidity and mortality by JUNV, no approved vaccines are currently available for other pathogenic arenaviruses. Among OW arenaviruses, Lassa fever virus is the most prevalent with over 300,000 infections and several thousand deaths occurring annually in Africa [69]. However, it is still a neglected tropical disease lacking any preventive vaccine or specific chemotherapy. Data presented here along with the reported effectiveness of **NSC4492** against the prototype OW LCMV suggest the interesting perspective on evaluating the activity of this disulfide against both OW and NW pathogenic arenaviruses. Further work on the protective efficacy of treated virions in an animal model will be required to validate the possible usage of **NSC4492** as an inactivating vaccinal compound.
